# *Tinospora cordifolia* ameliorates anxiety-like behavior and improves cognitive functions in acute sleep deprived rats

**DOI:** 10.1038/srep25564

**Published:** 2016-05-05

**Authors:** Rachana Mishra, Shaffi Manchanda, Muskan Gupta, Taranjeet Kaur, Vedangana Saini, Anuradha Sharma, Gurcharan Kaur

**Affiliations:** 1 Medical Biotechnology Laboratory, Department of Biotechnology, Guru Nanak Dev University, Amritsar, Punjab-143005, INDIA

## Abstract

Sleep deprivation (SD) leads to the spectrum of mood disorders like anxiety, cognitive dysfunctions and motor coordination impairment in many individuals. However, there is no effective pharmacological remedy to negate the effects of SD. The current study examined whether 50% ethanolic extract of *Tinospora cordifolia* (TCE) can attenuate these negative effects of SD. Three groups of adult Wistar female rats - (1) vehicle treated-sleep undisturbed (VUD), (2) vehicle treated-sleep deprived (VSD) and (3) TCE treated-sleep deprived (TSD) animals were tested behaviorally for cognitive functions, anxiety and motor coordination. TSD animals showed improved behavioral response in EPM and NOR tests for anxiety and cognitive functions, respectively as compared to VSD animals. TCE pretreatment modulated the stress induced-expression of plasticity markers PSA-NCAM, NCAM and GAP-43 along with proteins involved in the maintenance of LTP i.e., CamKII-α and calcineurin (CaN) in hippocampus and PC regions of the brain. Interestingly, contrary to VSD animals, TSD animals showed downregulated expression of inflammatory markers such as CD11b/c, MHC-1 and cytokines along with inhibition of apoptotic markers. This data suggests that TCE alone or in combination with other memory enhancing agents may help in managing sleep deprivation associated stress and improving cognitive functions.

Sleep as a physiological phenomenon has been found to play important role in improving and strengthening the functioning of the nervous, muscular and immune system, leading to accentuated growth and rejuvenation. Sleep has central role in learning and memory consolidation^1^. Currently, busy lifestyle, emotional imbalance, psychiatric disturbances and aging have resulted in sleep deprivation and related neurological disorders that consume a large fraction of health care resources. Regular sleep curtailment increases risk of metabolic disorders accompanied by hormonal imbalance thus, resulting in poorly regulated appetite, anxiety, stress, depression, emotional imbalance and obesity/diminished glucose tolerance^2^,^3^. Acute SD has been reported to induce serum level of Neuron specific enolase and S100- Ca^2+^ binding protein B^4^ and also 8-isoprostane (a marker of oxidative stress) concentration in plasma^5^. The oxidative damage caused by SD in the brain resulted in anxiety-like behavior and depression, which further induced avoidance behavior and delayed reaction times in various memory test^6^,^7^. Although, almost whole brain is affected by SD in one or the other way, the main areas that govern memory i.e., hippocampus and cerebral cortex are severely affected by SD and resulting anxiety and stress. Since, during sleep period, proteins involved in long term potentiation (LTP) stabilization are synthesized, hence, it has been proposed that the deleterious effect of SD on the cognitive functions is the result of its alteration in LTP in the hippocampus and cortex regions^8^,^9^.

In Ayurveda *T. cordifolia* has been kept under the category of Medha rasayana that helps in improving memory and cognition. Along with its radio- and chemoprotective effects, *T. cordifolia* has also been reported to show neuroprotective potential by modulating anti-oxidant enzyme system of brain tissue^10^. Oral dose of ethanolic extract of *T. cordifolia* has been found to enhance the dopamine level and complex-I activity in 6-hydroxy dopamine (6-OHDA) induced Parkinson model of rat along with reduced oxidative stress and improved locomotor activity^11^. Petroleum ether extract of *T. cordifolia* has been reported to reverse the depression-like behavior in mice and reduced the monoamine oxidase activities in the brain, resulting in increased levels of brain monoamines^12^. Extract of *T. cordifolia* has been further shown to modulate expression of pro-inflammatory cytokines along with COX-2, and iNOS in other inflammatory diseases like asthma, edema and resulting in reduced hyper-responsiveness^13^,^14^.

The present study was aimed to explore the effect of 50% ethanolic extract of *T. cordifolia* stem (TCE) in ameliorating anxiety-like behavior induced in acute SD rats and resulting cognitive deficit and motor coordination. We further elucidated the underlying molecular basis of these behavioral effects, by studying the expression of proteins involved in synaptic plasticity, neuroinflammation and cell survival signaling in hippocampus and pyriform cortex regions of SD animals.

## Results

### TCE suppressed the anxiety-like behavior in SD animals and improved exploratory behavior

**Figure 1:**
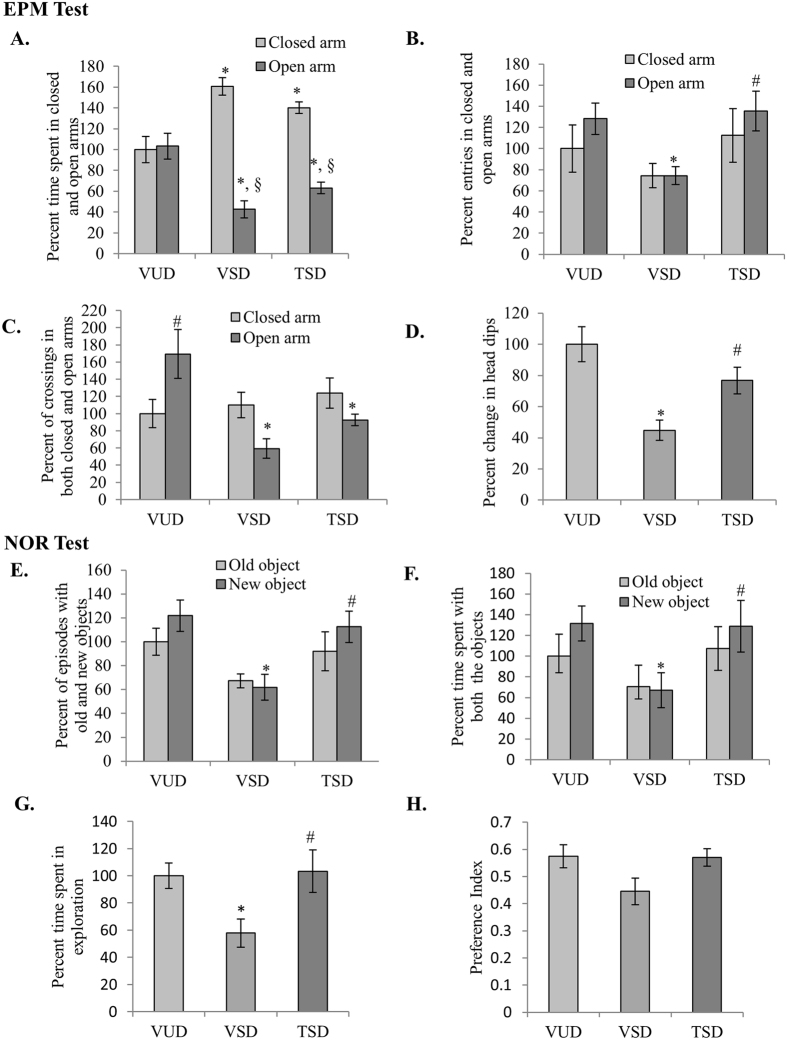
TCE improved exploratory behavior of SD animals. In EPM test sleep deprived animals spent more time in closed arm (**A**) however TSD animals spent more time in exploration as indicated by higher number of entries and crossings (**B**,**C**). TSD group showed suppressed anxiety-like behavior and had higher number of head dips as compared to VSD (**D**). In NOR test TSD animals showed almost similar behavior as VUD animals as indicated by higher number of episodes for each object as compared to VSD animals (**E**). TSD group spent more time in exploring each object as compared to VSD animals (**F**,**G**). However there is not much difference in preference index for each object among the three groups (**H**). ‘*’ represent statistical significant difference *p* < 0.05 as compared to VUD group, ‘**#**’ represent statistical significant difference *p* < 0.05 as compared to VSD animals and ‘**§**’ represent statistical significant difference within the group.

Animals, while testing on EPM for anxiety-like behavior, were not given any prior training to study their response for spatial novelty. Among the groups, both VSD animals spent significantly lesser time in open arm and more time in closed arm as compared to VUD animals (F_(2, 46)_ = 21.675, p ≤ 0.001) followed by TSD animals as compared to VUD animals (Fig. 1A). Further, all the three groups showed significant difference in the number of entries in the arms (F_(2, 46)_ = 1.225, p = 0.011). VSD animals showed significantly lower number of entries in open arms as compared to VUD animals (p ≤ 0.05) but they did not show any significant difference in case of closed arm entries. However, TSD animals showed higher activity as compared to VSD animals as their number of entries was similar to VUD animals (Fig. 1B). Among the groups, VSD animals showed significantly lower number of crossings in open arm as compared to VUD animals, which was followed by TSD animals (F_(2, 46)_ = 7.323, p = 0.002) (Fig. 1C). TSD animals showed more number of entries and crossings in closed and open arms as compared to VSD group animals indicating their improved exploratory behaviour. Further, VSD animals also showed statistically significantly lower number of head dips as compared to VUD animals (p ≤ 0.05), which is marker of anxiety in rodents. However, TSD animals showed increased number of head dips, indicating lower level of anxiety (Fig. 1D).

We further tested these animals for Novel Object Recognition (NOR) test. Among the groups, VSD animals showed lower number of episodes for both old and new object as compared to VUD and TSD animals (F_(2, 46)_ = 8.149, p = 0.001) (Fig. 1E), whereas, both VUD and TSD animals showed significantly higher number of episodes with new object as compared to old (p ≤ 0.05). Further, VSD group animals spent significantly less time in exploration of both the objects, whereas TSD animals showed almost similar behavioral pattern as VUD animals and spent more time in explorating the objects (F_(2, 46)_ = 5.999, p = 0.005). Similar to VUD animals, TSD group animals spent significant more time with new object as compared to VSD animals (p ≤ 0.05) (Fig. 1F). Although, we did not find any significant difference in preference index among the three groups but VSD animals showed preference index below 0.5 showing their least preference for novel object, whereas, TSD group animals showed preference index similar to VUD animals between 0.5–0.6 showing their preference of novel object (Fig. 1H).

**Figure 2:**
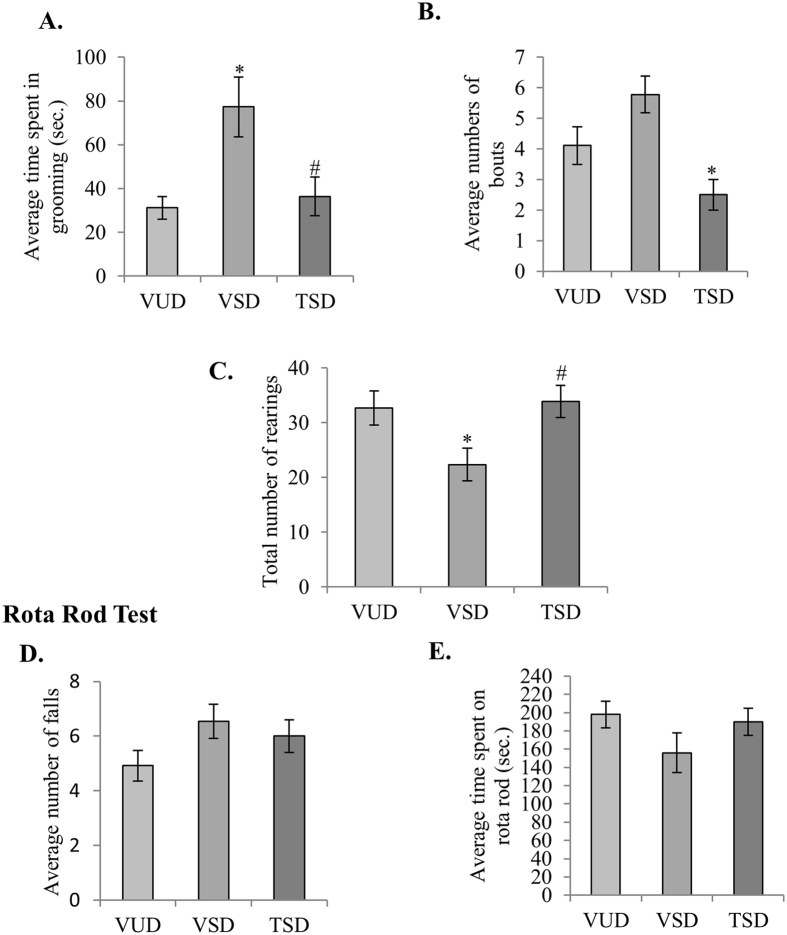
TCE suppressed the anxiety-like behavior in SD animals. Anxiety like behavior was shown to reduce in TSD animals as they spent less time in grooming (**A**) and showed significantly less number of grooming bouts as compared to VSD animals (**B**). Further, rearing behavior in TSD animals was found to be comparable to VUD animals, which was higher than VSD animals (**C**). There was no significant change in motor coordination in the three groups animals tested on rotarod (**D**,**E**). ‘*’ represent statistical significant difference *p* < 0.05 as compared to VUD group and ‘**#**’ represent statistical significant difference *p* < 0.05 as compared to VSD animals

Rodents are known to groom themselves in both high and low stress conditions. Thus, we studied grooming behavior along with the novel object recognition test to correlate effects of SD-induced stress and anxiety on memory and cognition. VSD animals showed slightly higher number of grooming bouts as compared to VUD animals and spent statistically significant more time in grooming (p ≤ 0.05) (Fig. 2A). Whereas, grooming behavior in TSD animals was significantly reduced as compared to VSD animals as indicated by significantly reduced number of grooming bouts and time spent in grooming (Fig. 2B). Further, TSD animals also showed significantly more rearing as compared to VSD animals (p ≤ 0.05) (Fig. 2C). The data may suggest that TCE has the potential to suppress the anxiety-like behavior in SD animals and improve their exploratory behaviour.

In neuromuscular coordination test on rotarod, all the three groups animals did not show any significant difference for the number of falls and total time spent on rotarod (Fig. 2D,E).

### TCE modulated the expression of proteins involved in synaptic plasticity and LTP maintenance

**Figure 3:**
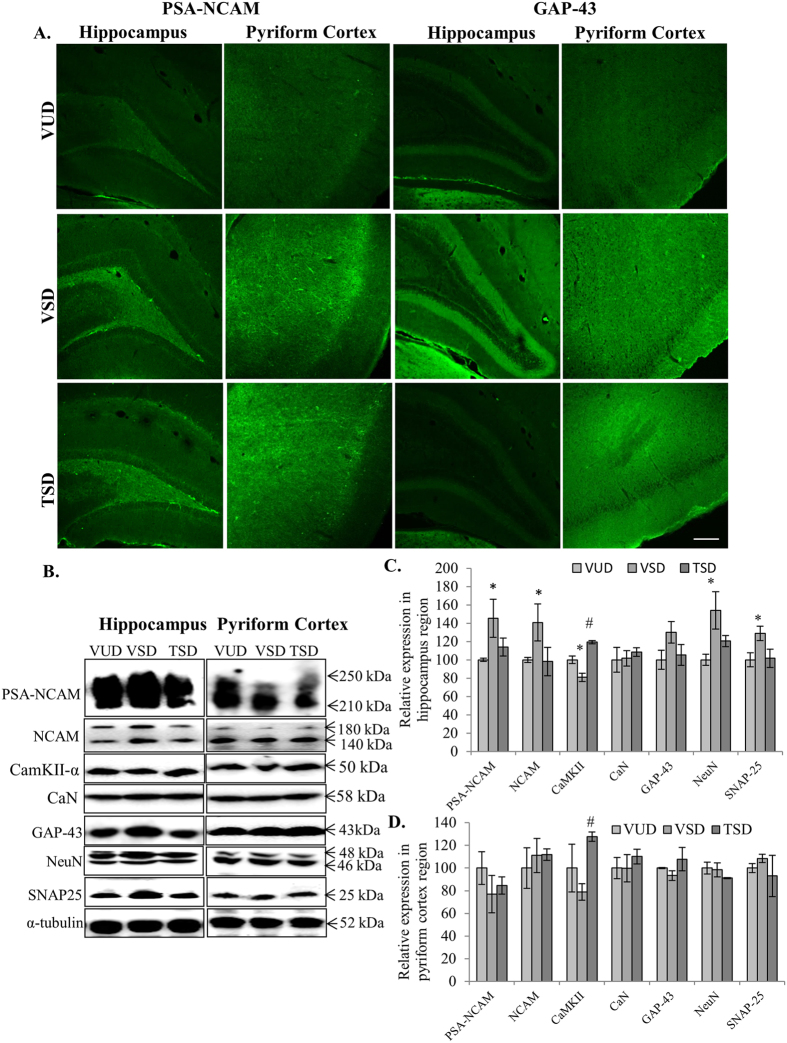
TCE modulated stress induced changes in expression of proteins involved in structural plasticity and LTP maintenance in acute sleep deprived animals. Immunohistochemical analysis of 35 μm thick coronal sections of brains showed stress induced expression of PSA-NCAM and GAP-43 in hippocampus and pyriform cortex regions in VSD and TSD animals as compared to VUD animals. Scale bar- 200 μm (**A**). Further, western blot analysis showed enhanced expression of PSA-NCAM, NCAM, GAP-43, NeuN and SNAP-25 on sleep deprivation especially in hippocampus region which was modulated by TCE treatment. However, expression of CaMKII-α was found to be downregulated in VSD animals which was further induce in TSD animals (**B**). Histograms represent normalized relative densitometric values of these markers obtained from three different experiments and plotted as mean ± SEM for hippocampus and pyriform cortex region respectively (**C**,**D**). ‘*’ represent statistical significant difference *p* < 0.05 between VUD and test groups, whereas, ‘#’ represent statistical significant difference *p* < 0.05 between VSD and TSD group.

Change in expression of proteins involved in synaptic plasticity and LTP maintenance in brain is the primary reaction against any type of stress. As hippocampus and pyriform cortex are the regions associated with memory and cognition and also most susceptible for degenerative processes associated with SD, we examined the effect of acute SD on the expression of these proteins in these discrete regions of the brain. Expression of PSA-NCAM was found to be significantly upregulated in hippocampus region of VSD animals as compared to VUD animals (p ≤ 0.05). Whereas, in TSD animals, only slight increase in PSA-NCAM was observed in hippocampus as compared to VUD animals (Fig. 3A,B first panel and C). The increase in PSA-NCAM expression in hippocampus region of VSD animal brain was accompanied by the enhanced expression of both 140 and 180 kDa isoforms of NCAM (p ≤ 0.05) (Fig. 3B second panel and C). Among the three groups, there was no significant change in expression of PSA-NCAM and NCAM in PC region. We further studied expression of Ca^2+^ -dependent/calmodulin proteins CaN and CaMKII-α in the hippocampus and PC region of the entire three groups. CaMKII-α plays critical role in LTP induction and in coordination with CaN, maintains LTP in discrete brain regions. VSD animals showed decreased expression of CaMKII-α in hippocampus (p ≤ 0.05) as well as PC region as compared to VUD animals. However, TSD group showed significant increase in expression of CaMKII-α in both regions (p ≤ 0.05) (Fig. 3B third panel and C). We did not detect any significant change in expression of CaN in both the brain regions of all the three animal groups (Fig. 3B fourth panel, C and D).

Further, VSD animals also showed enhanced expression of proteins which are specific for mature neurons and least expressed in proliferating cells i.e., GAP-43 and NeuN especially in hippocampus region of VSD animals as compared to VUD animals, which was suppressed in TSD animals (Fig. 3A,B fifth panel and sixth panel and C). A key component of SNARE complex, SNAP-25 is critical for coordinated functioning of Ca^2+^ influx and fusion of vesicle containing neurotransmitters. In the present study, VSD animals showed significant increase in expression of SNAP-25 protein as compared to its baseline expression in VUD group in hippocampus region (p < 0.05). However, TSD group did not show any change in its expression (Fig. 3B seventh panel, C and D). Optimum expression of SNAP-25 is essential for structural flexibility of SNARE complex and for both facilitated and non-facilitated neural transmission. In PC region we did not detect any significant changes in these proteins.

### TCE inhibited glial cell activation and neuroinflammation

**Figure 4:**
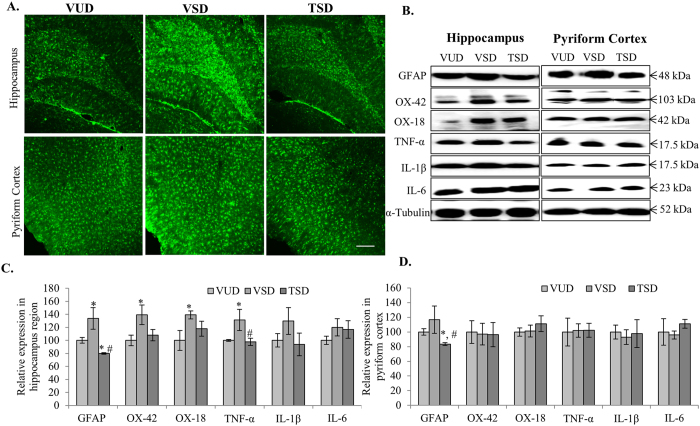
TCE suppressed activation of glial and microglial cells along with inflammatory cytokines, induced by acute sleep deprivation. Immunohistochemical analysis of 35 μm thick coronal sections of brains showed reduced expression of GFAP in both hippocampus and cortex regions in TSD animals which was found to be upregulated in the same regions in VSD animals as compared to VUD. Scale bar- 200 μm (**A**). Further, western blot analysis showed reduced level of GFAP, iC3b complement receptor identified by OX-2 antibody, MHC-1 molecule identified by OX-18, TNF-α, IL-1β and IL-6 in hippocampus and PC regions in TSD animals as compared to VSD animals (**B**). Histograms represent normalized relative densitometric values of these markers obtained from three different experiments and plotted as mean ± SEM for hippocampus and pyriform cortex region respectively (**C**,**D**). ‘*’ represent statistical significant difference *p* < 0.05 between VUD and test groups, whereas, ‘#’ represent statistical significant difference *p* < 0.05 between VSD and TSD group.

Glial cells play important role in neuronal resilience. However, they are also key mediators of immune response in various brain diseases. SD of 12 h in VSD animals induced the expression of astroglial cell specific protein GFAP (p ≤ 0.05) (Fig. 4A,B first panel, C and D). SD induced astroglial cell activation was further accompanied by activation of microglial cells as indicated by over expression of microglial cell specific markers integrin alpha-M i.e., CR-3 and CR-4 (detected by OX-42 antibody) along with MHC-1 molecule (detected by OX-18 antibody) especially in hippocampus region, as compared to VUD animals (p ≤ 0.05) (Fig. 4B second and third panel, C and D). These are key components of immune response and leads to activation of inflammatory pathways. TCE pretreatment was observed to suppress the expression of these astroglial and microglial activation markers (p ≤ 0.05) as compared to VSD animals.

Activation of glial cells ultimately results in reactive gliosis and activation of inflammatory pathways in a cyclic manner. Thus, we further studied the expression of inflammatory cytokines that are well known mediators of neuroinflammation in chronic diseases. Interestingly, VSD group showed upregulated expression of IL-1β and IL-6 with significant increase in TNF-α especially in hippocampus region (p ≤ 0.05) as compared to VUD animals (Fig. 4B fourth, fifth and sixth panel and C). However, there was no significant change in expression of these inflammatory cytokines in the discrete brain regions of TSD group as compared to VUD animals. This data may suggest that acute SD primarily affects hippocampus region of the brain by inducing activation of glial/microglial cells which subsequently lead to activation of inflammatory cytokines while, TCE pretreatment may inhibit the activation of these cells.

### TCE modulated mitochondrial stress response proteins and inhibited apoptosis

**Figure 5:**
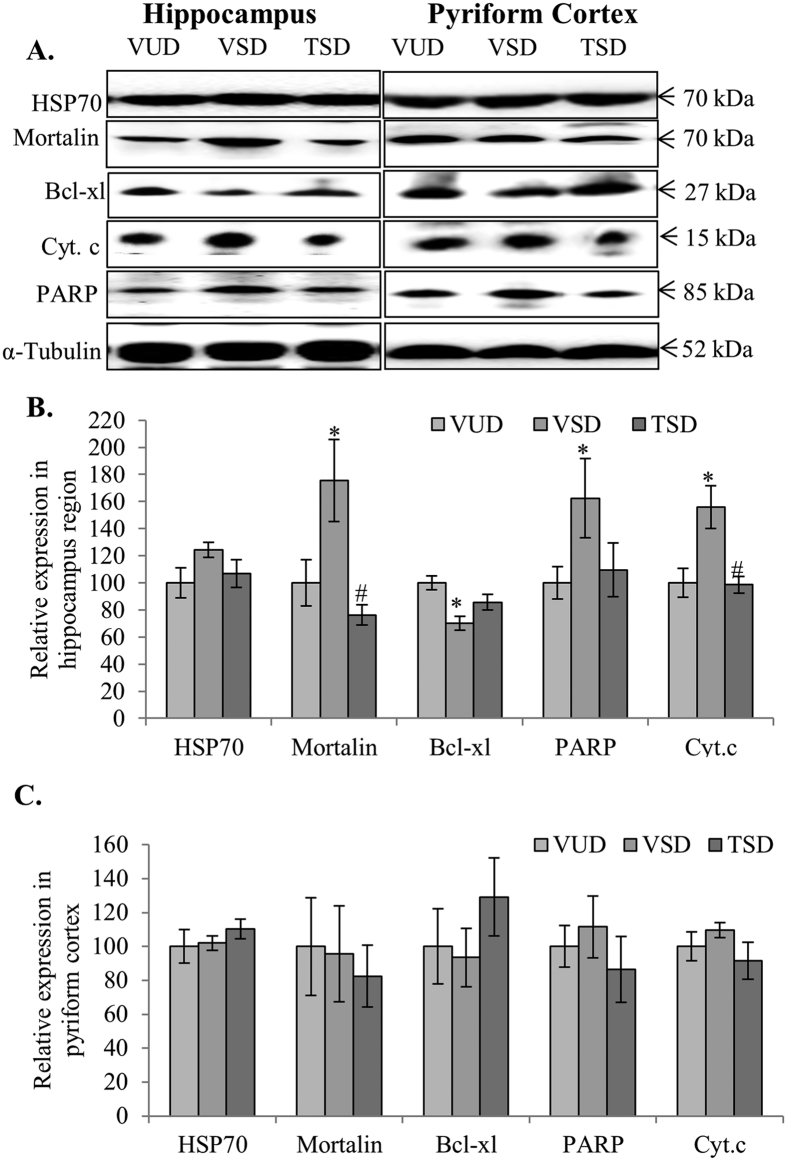
TCE reduced oxidative stress and apoptosis induced due to acute sleep deprivation. Western blot analysis for HSP-70, mortalin, cyt. c, and PARP in hippocampus and pyriform cortex regions of the three group showed induced expression of these markers and reduced expression of bcl-xl in hippocampus and pyriform cortex regions in VSD animals as compared to VUD animals which was found to be further modulated in TSD animals (**A**). Histograms represent normalized relative densitometric values of HSP-70, mortalin, bcl-xl, cyt. c, and PARP obtained from three different experiments and plotted as mean ± SEM for hippocampus and pyriform cortex region respectively (**B**,**C**). ‘*’ represent statistical significant difference *p* < 0.05 between VUD and test groups, whereas, ‘#’ represent statistical significant difference *p* < 0.05 between VSD and TSD group.

Molecular chaperones like stress marker HSP-70 and cell senescence marker mortalin play important role in mitochondrial dynamics. As molecular chaperones are involved in protein folding and unfolding and their transportation within the cell organelles, they are primary response elements to any oxidative stress in mitochondria. VSD animals showed constant but non-significant increase in both HSP-70 and mortalin in hippocampus region as compared to VUD animals. However, TSD group showed almost similar expression of these chaperone proteins as VUD animals (Fig. 5A first and second panel, B and C). The current data is in line with the reports suggesting that extended wakefulness induces oxidative stress in the brain along with activation of inflammatory pathways, ultimately leading to apoptosis. Thus, we further examined the expression of anti-apoptotic protein bcl-xl and its downstream molecules cyt. c and PARP, in both hippocampus and PC regions of the brain in all the three test groups. Interestingly, VSD animals showed significant downregulation in bcl-xl (p ≤ 0.05), which was further accompanied by increased expression of cyt. c and 85 kDa fragment of PARP (a classical marker of apoptosis) in hippocampus (p ≤ 0.05) as well as PC regions, as compared to VUD animals (Fig. 5A third, fourth and fifth panel, B and C). Reduced expression of bcl-xl and increased expression of cyt. c and PARP make a ‘point of no return’ during apoptosis. However, TSD animals showed bcl-xl expression similar to VUD animals in both hippocampus and PC regions resulting in reduced apoptosis which was evident from the significantly downregulated expression of cyt. c and PARP as compared to VSD animals (p ≤ 0.05) (Fig. 5A fourth and fifth panel, B and C).

### TCE modulated cell survival proteins

**Figure 6:**
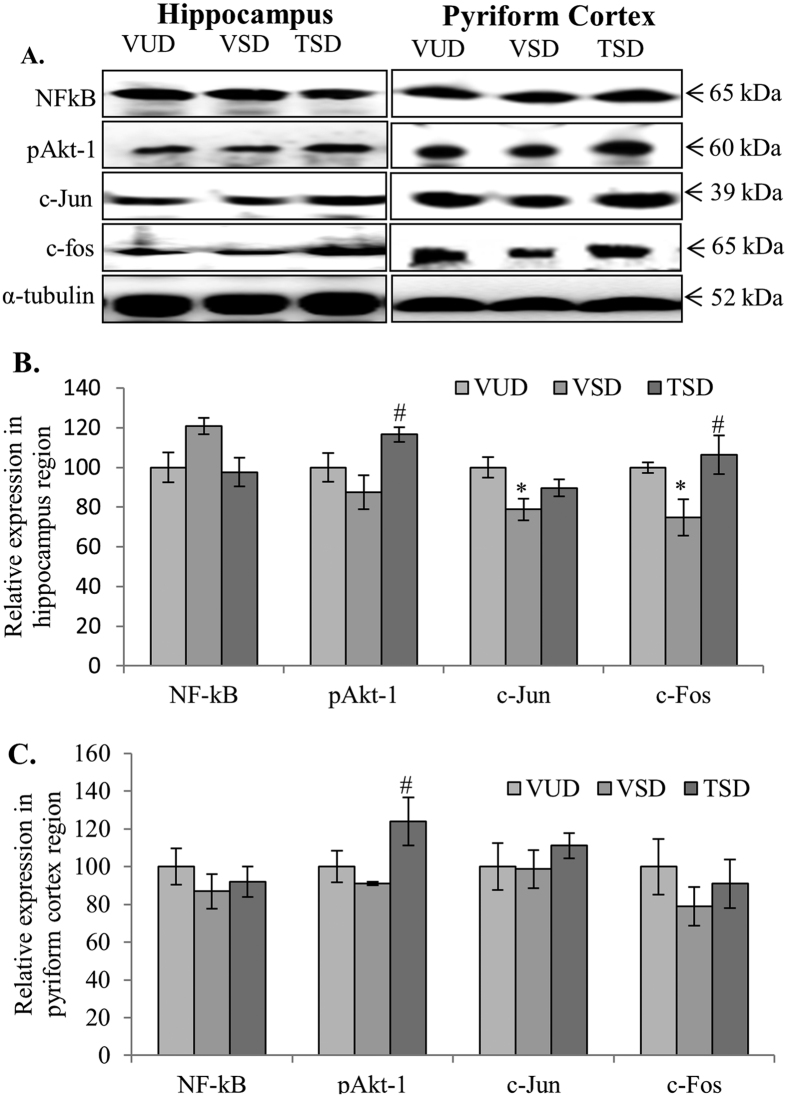
TCE induced cell survival pathways in acute sleep deprived animals. TSD animals showed reduced NF-κB but induced expression of pAkt-1, c-Jun and c-Fos in hippocampus and pyriform cortex regions as compared to VSD animals (**A**). Histograms represent normalized relative densitometric values of NF-κB, pAkt-1, c-Jun and c-Fos obtained from three different experiments and plotted as mean ± SEM for hippocampus and pyriform cortex region respectively (**B**,**C**). ‘*’ represents statistical significant difference *p* < 0.05 between VUD and test groups, whereas, ‘#’ represent statistical significant difference *p* < 0.05 between VSD and TSD group.

Since, TCE was observed to suppress apoptosis, it was further planned to study whether TCE also has any effect on the expression of molecular markers associated with cell survival. VSD animals showed a constant but non-significant increase in expression of NF-κB in hippocampus region as compared to VUD animals (Fig. 6A first panel and B). Whereas, TSD group of animals did not show any change in NF-κB expression. No significant change was seen in expression of NF-κB in PC region among all the three animal groups. NF-κB plays dual role to promote cell survival as well as induce expression of inflammatory cytokines. Further, Akt-1 is an important molecule that plays key role in neuritogenesis, axonal growth and signaling. Its phosphorylation at serine-473 is important for various molecular interactions involving cell survival signaling. Western blot analysis indicated reduced level of pAkt-1 (pSER473) in both hippocampus and PC regions of the brain of VSD animals as compared to VUD group (Fig. 6A second panel, B and C). However, TCE pretreatment to SD animals significantly enhanced the level of pAkt-1 as compared to VSD animals (p ≤ 0.05).

Reduced level of p-Akt-1 in VSD animals was also accompanied by downregulation in expression of ‘immediate early genes’ (IEGs) i.e., c-jun and c-fos at translational level which are actively involved in the induction of cell survival and maintenance of homeostasis of the cellular environment (Fig. 6A third and fourth panel, B and C). However, TSD group showed increase in expression of c-jun and c-fos in hippocampus as well as PC region as compared to VSD animals. This data may suggest that TCE treatment induced survival signaling in the SD animals which may have resulted in reduced apoptosis and oxidative stress.

## Discussion

The current study provides first ever evidence that 50% ethanolic extract of *Tinospora cordifolia* (TCE) possesses anxiolytic properties and may play important role in preventing anxiety induced learning and memory impairments. In the present study, 12 h SD regimen was used which is approximately equivalent to “one night sleep deprivation in humans’. SD of 12 h was found to induce anxiety like behavior in VSD animals. Grooming is the first behavioral response of rodents to both high and low stress conditions^15^,^16^. Rodents generally follow a sequential grooming pattern starting from rostrum up to tail, which is interrupted in anxious animal. In present study also, VSD animals showed increased grooming with interrupted pattern. This is in line with the other stress related studies which have reported enhanced grooming behavior in several SD paradigms^17^,^18^. Enhanced grooming behavior was also accompanied by lower number of head dips and less rearings as compared to control group animals. However, TSD group showed higher number of head dips, more rearings and lesser grooming bouts. Rodents showing more rearings have been found to be less stressed and more explorative. Further, despite spending significantly more time in the closed arm during the classical test of anxiety i.e., EPM, TSD animals remained active and explorative as indicated by higher number of crossings and entries in both the arms. Whereas, VSD animals spent most of their time in closed arm but without any major activity. This suggests that 15 days of TCE administration to these animals prior to SD ameliorate the anxiety-like behavior and also restored the exploratory behavior of these animals.

Various studies and clinical data have shown direct link between anxiety and cognitive impairment or avoidance behavior in both animal and humans^6^,^7^. Thus, we tested all the three animal groups for NOR. The data indicated improved exploratory behavior in TSD group animals as they spent significantly more time in exploring both the objects with more number of total episodes for each object, as compared to VSD group. Although, we did not find any significant difference in the preference index (PI), but VSD animals did not show preference for novel object (PI ≤ 0.5). This may be due to unwillingness of the VSD animals for exploring both the objects whether new or old. However, TSD group showed preference for novel object similar to VUD animals (PI ≥ 0.5). It may be suggested that the improved behavioral response in TSD animals may be the result of reduced anxiety, higher activity level and improved cognitive functions. Acute sleep deprivation of 12 h did not seem to affect the neuromuscular coordination of the animals as none of the groups showed any significant difference for the number of falls and total time spent on the rotarod.

Further experiments to discern the underlying molecular mechanism for the differences in the behavioral trends for all the three test groups revealed enhanced expression of synaptic plasticity markers PSA-NCAM and NCAM in VSD group. Despite, being the key molecules for regulating structural plasticity in brain regions like hippocampus and pyriform cortex^19^ (which are associated with learning and memory behavior), their expression has been found to be upregulated under various stress conditions and mood disorders, including juvenile^20^ and chronic stress like restraining^21^. As VSD animals showed enhanced PSA-NCAM and NCAM expression in hippocampus as compared to VUD group, this increase may be the result of compensatory mechanisms to overcome the stress and to retain the structural and functional integrity and neural networking. However, TCE pre-treatment to the SD animals may have reduced the cellular stress thus inhibited the stress induced increase in the expression of PSA-NCAM and NCAM. These results are in line with the study by Higuera-Matas *et al*.^22^, who reported that cannabinoid treatment of adolescent rats caused working memory impairment and also significantly enhanced the expression of PSA-NCAM and NCAM without changing LTP^22^.

Further, VSD group showed significant downregulation in CaMKII-α expression in hippocampus as well as PC regions while expression of CaN was not altered (Fig. 3). Serine/threonine protein kinase CaMKII-α and protein phosphatase CaN are the two most instrumental proteins in regulation of Ca^+2^-dependent synaptic plasticity, release of neurotransmitters, learning and memory and play critical role in maintenance of LTP^23^,^24^. Various other studies also indicated that CaMKII-α expression and its activation are impaired in hippocampus after SD of varied duration while activity of CaN was increased without changing its base line expression^25^,^26^. Interestingly, TCE pre-treatment in SD animals enhanced the expression of CamKII-α in both hippocampus and PC regions. Decrease in expression of CaMKII-α may be one of the important factors for the poor performance of SD animals in novel object recognition test, which was corrected by TCE pretreatment in hippocampus region and improved learning and memory. Further, VSD animals also showed upregulated expression of SNARE complex protein SNAP-25 which is highly involved in synchronizing neurotransmission through highly dynamic and coordinated mechanism of Ca^2+^ influx and neurotransmitter vesicles infusion with cell membrane^27^. Interestingly, SNAP-25 knockouts showed decreased NMDARs expression at synapses impairing LTP induction^28^; however, its overexpression has been shown to enhance sensorimotor gating defects and depression-like behavior^29^. Optimum expression of SNAP-25 is essential for structural flexibility of SNARE complex and for both facilitated and non-facilitated neural transmission. SD of 72 h especially in hippocampus and prefrontal cortex region has been reported to up-regulate SNAP25 expression^30^. In the present study, TCE pre-treatment normalized the expression of SNAP-25 and induced the expression of CamKII-α in SD animals especially in hippocampus region (Fig. 3), which may have resulted in improved cognitive response in TSD animals.

Along with modulation of synaptic plasticity and LTP maintenance proteins, VSD animals also showed enhanced expression of GAP-43 and NeuN which are markers for mature neurons and least expressed in proliferating cells^31^,^32^. Phosphorylated form of GAP-43 along with various other cytoskeletal proteins, including α-tubulin, F-actin, neurofilament protein and RhoGTPase have been reported to be upregulated after acute SD^33^. It is well reported in literature that disturbed sleep in any form reduces neurogenesis in the hippocampus region^34^. SD caused reduction in proliferating neuronal cells as indicated by Brdu/NeuN staining^35^. However, in TSD group its expression was found to be comparable to VUD group, which supports potential beneficial role of TCE in brain plasticity. The data also indicates that acute SD-associated stress/anxiety altered the expression of proteins involved in structural plasticity especially in hippocampus region of the brain.

To further evaluate the effect of anxiety-like behavior on the glial and microglial cells we studied expression of various molecular markers specific for these cells along with inflammatory cytokines. 12 h SD induced activation of astroglial and microglial cells in VSD group as indicated by up-regulated expression of GFAP (astroglial marker), integrin alpha-M (the receptor for component of complement i.e., CR-3 and CR-4) along with MHC class-1 molecule especially in hippocampus region as compared to control VUD group (Fig. 4). Various studies with short term SD have reported up regulation of GFAP expression in hippocampus after 4 and 24 h of SD^36^,^37^. Further, activation of these glial cells in VSD group was also accompanied by non-significant upregulated expression of NF-κB and its downstream inflammatory cytokines such as TNF-α, IL-1β and IL-6 in hippocampus. The complex and context dependent expression of NF-κB shows dramatically opposite results, such as its activation in neuronal cells promotes their survival while its cyclic activation in astroglial and microglial cells mediates pathological inflammatory reactions^38^. Microgliosis and gliosis are indicative of their involvement in the deleterious effect of short as well as long term SD. A plethora of research evidence suggests link between SD-induced IL-1β, IL-6 and TNF-α expressions to SD associated disorders^39^,^40^,^41^. Thus, it is suggested that excessive secretion of these inflammatory molecules may have resulted the activation of astroglial and microglial cells and also contributed to anxiety like behavior in VSD animals. However, TCE pretreatment suppressed the expression of GFAP, integrin alpha- and MHC-1 class molecules along with NF-κB and inflammatory cytokines, thus depicting the neuro-immunomodulatory activity of TCE. It may be suggested that TCE may have inhibited the induction of these inflammatory responses thus protecting the discrete brain regions under acute stress conditions and contribute to the suppression of the anxiety-like behavior. Extract of *T. cordifolia* has been recently reported to modulate expression of these pro-inflammatory cytokines along with other mediators of inflammation such as COX-2, and iNOS in other inflammatory diseases like asthma, edema resulting in reduced hyper-responsiveness^14^,^15^.

One of the important functions of sleep is to reduce mitochondrial oxidative stress which is generated during extended wakefulness^42^. Further, in developing brain active sleep plays key role in inhibiting apoptosis^43^. Various recent reports have also shown induction of apoptosis after REM sleep deprivation^44^,^45^. In the present study, VSD group showed higher mitochondrial stress as compared to VUD group, as evident from the enhanced expression of stress marker HSP-70. Senescence marker mortalin along with apoptotic marker protein PARP-1(85 kDa) was also significantly upregulated which may have resulted in enhanced release of cyt. c (Fig. 5). Stress-associated induction of these chaperone proteins is known to protect cells against the initial insult and helps in improving cell recovery. However, under high stress conditions HSP-70 has been reported to induce expression of IL-6 and TNF-α in microglial cells resulting in increased phagocytic activity^46^, which may explain the changes observed in VSD animals. Further, 116 kDa PARP cleavage into 85 and 25 kDa fragment has been considered as a classical characteristic of apoptosis. This increase in apoptosis in VSD animal brain may be the result of decreased expression of anti-apoptotic protein bcl-xl and increased expression of NF-κB. Activation of pro-apoptotic/apoptotic pathway through activation of inflammatory cytokines by NF-κB, causes Bax/Bak-dependent mitochondrial membrane permeabilization and activation of caspases^47^. Interestingly, in TSD group, we detected reduced expression of HSP70 and mortalin and significant upregulation of anti-apoptotic protein bcl-xl along with decreased level of cleaved PARP (85 kDa fragment) and cyt. c. Decreased expression of bcl-xl is known to induce massive apoptotic cell death in developing neurons throughout nervous system^48^. However, upregulated expression of bcl-xl has been found to protect hippocampal and cortical neuronal cells^49^. It may be suggested that TCE has the potential to reduce the mitochondrial stress and inhibit the expression of proteins involved in mediating apoptosis by reducing activation of glia and microglial cells.

REM sleep deprivation has been shown to reduce phosphorylation of Akt^50^. In the present study, the induction of apoptosis in VSD animals may also be the result of decreased level of pAkt-1 as pAkt-1 inhibits PARP cleavage and via activation of PI3K and inhibition of apoptosis inducer PTEN, leads to cell survival and inhibition of apoptosis^51^,^52^. Further, Akt activation via phosphorylation of serine-473 residue has very important role in neuroprotection, growth, regulation of cell differentiation, and neurite outgrowth, including elongation and branching through PI3K/Akt signaling pathway^53^. We observed that as compared to VSD animals, TSD group animals showed higher level of pAkt and decreased PARP (85 kDa fragment). This may indicate that the cell survival and inhibition of sleep deprivation induced apoptosis in TSD group animals may be the result of induction of pAkt pathway. Various neuroprotective agents like Bisperoxovanadium (BpV), a tyrosine phosphatase inhibitor of PTEN, also acts via activation of Akt/PI3K pathway^54^. Interestingly, pAkt in coordination with CamKII is also known to help in maintenance of LTP^55^. TCE pretreatment in SD animals increased the levels of both pAkt-1 and CamKII-α indicating the neuroprotective potential of this plant extract.

**Figure 7:**
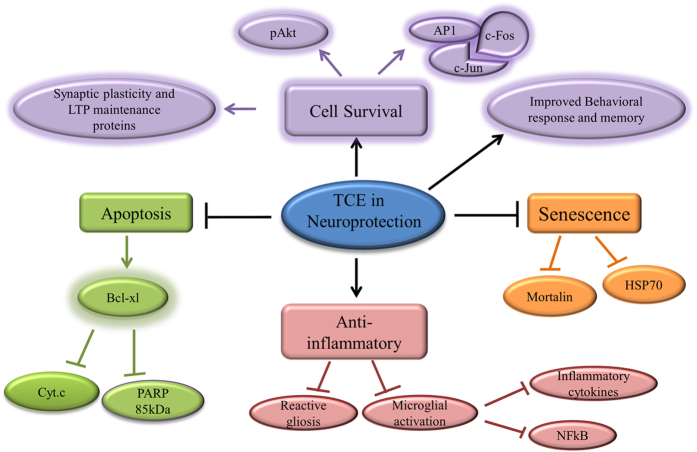
Graphical Abstract: underlying molecular pathways in the neuroprotective activity of TCE.

Unlike VSD group, TCE treated animals were further observed to show upregulation of other cell survival pathway proteins like c-fos and c-jun, in both hippocampus and cortical regions of the brain (Fig. 6). c-Fos and c-jun are categorized under ‘IEGs’ and their expression has been found to be upregulated by several stimuli. c-Fos was found to be a critical component in conditioned taste aversion learning, however, c-jun is important for c-fos activity in consolidation process^56^,^57^. The expression of these ‘IEGs’ has been found to be maximum after SD up to 3 h which then declines progressively^58^. However, chronic sleep fragmentation leads to decreased expression of c-fos in wake-active neurons^59^. Thus, decreased c-fos expression in hippocampus and cortical region of the brain of VSD animals after 12 h of SD may be correlated with their slow responsiveness and activity in behavioral tests, whereas, TSD animals with enhanced expression of cell survival proteins showed improved activity which was comparable to VUD group. Thus, TCE seems to inhibit stress induced changes in the two discrete brain regions along with inhibiting activation of apoptotic pathways by inducing cell survival (schematic representation of possible molecular pathways in TCE activity in sleep deprived animals in Fig. 7).

As sleep provides a time window to facilitate undisturbed memory formation and consolidation, any disturbance in sleep whether acute or chronic may lead to impairment of memory and cognition. However, various professional services like military, medical interns, and pilots etc. demand high attentiveness and accurate mental performance, even after prolonged wakefulness. Under such situations, the ability to adapt and attention to the spatial context and references determines the performance of the person which is mainly regulated by hippocampus in coordination with cerebral cortex. Considering the acute effects of SD, Ayurvedic medicines including *T. cordifolia* may provide safe and effective therapeutics for prevention of brain function impairments including Mild Cognitive impairment (MCI) associated with SD. The current data indicates that whereas long term sleep apnea affects both cerebral cortex and hippocampus^8^,^9^,^60^, acute SD of 12 h primarily affects only hippocampus region of the brain thus may be affecting hippocampus dependent memory formation only. It may further be suggested that consumption of extract of *T. cordifolia* alone or with other memory enhancing and anti-anxiety natural agents may help in managing mental stress and improving memory and cognition.

## Material and Methods

TCE was obtained from Indian Institute of Integrative Medicines, Jammu, India. Briefly, stem of T. cordifolia was shade dried and grinded to fine powder. This powder was then mixed with 50% ethanol and incubated at 37 °C with continuous shaking for overnight and filtered with Whatman I filter paper. The extract thus obtained was air dried and reconstituted in water at 100 mg/ml concentration for the oral feeding of the animals.

### Animals and extract administration

Wistar albino female rats in the age group of 11–12 months and weighing 150–170 g were caged in the group of three under controlled environment (constant temperature of 25 ± 2 °C and constant dark/light cycle of 12:12 h) with *ad libitum* food and water supply. All animal experimental protocols were approved by Institutional Animal Ethical Committee, Guru Nanak Dev University, Amritsar, India (permission number 226/CPCSEA-A/2014/37) and performed in accordance with the guidelines of ‘Animal Care and Use’ laid down by Institutional Animal Ethical Committee, Guru Nanak Dev University.

Animals were grouped randomly, irrespective of their estrous cycle stage, to eliminate the effect of estrogen factor on their behavior, in 3 groups- 1. Vehicle Undisturbed sleep (VUD) 2. Vehicle Sleep Deprived (VSD) and 3. TCE treated Sleep Deprived (TSD) with 9–10 animals per group. VUD and VSD group were given water as vehicle. Dried TCE was reconstituted in water and TSD Animals were given oral dose of 140 mg/kg of body weight of TCE between 9:00 to 10:00 AM for 15 days. In literature, researchers have used various doses of TCE (from 50 mg/kg to 300 mg/kg of body weight) for treatment in different *in vivo* experiments^12^,^13^,^14^. However, dose of aqueous ethanolic extract of *Tinospora cordifolia* between 100 mg/kg to 200 mg/kg of body weight have been found to be most effective. So, for the present study, dose of 140 mg/kg of body weight was selected on the basis of previous reports. On 15^th^ day VSD and TSD group were sleep deprived during light phase for 12 h from 6:00 AM to 6:00 PM by gentle handling method followed by behavioral studies. Animals were kept in their home cage and were monitored by the trained experimenter with whom they were familiarized prior to the start of actual experiment. For the total sleep deprivation the rats were gently stroked with a soft brush or their cages were gently shaked, just to keep them awake whenever, drowsiness or attempt to acquire a sleep posture was observed.

## Behavioral Test

### Elevated Plus Maze (EPM) test

On 15^th^ day of treatment, VSD and TSD groups were sleep deprived for 12 h and all the three groups animals were subjected to the EPM test which consisted of two opposing open (50 × 10 cm) and closed arms (50 × 10 × 50 cm) extending from the central platform (10 × 10 cm), crossing each other in the form of a plus, elevated 50 cm above the floor. At the beginning of the test, the individual rats were placed on the central platform facing one of the closed arms and were allowed to freely explore the plus maze for 5 min. Whole experiment was performed in dim light to encourage rats to explore open arms. The behavior of each rat was monitored using a video camera and their movements were registered and analyzed. The whole apparatus was thoroughly cleaned using 5% ethanol before placing next rat. An entry into any arm was defined as entry of all four limbs into that arm. The time spent by each rat in each arm was recorded. Crossing was defined as animals emerging from one arm and entering with all four paws, to its opposite arm. The behavioral analysis included measuring the frequency and duration of the following parameters: (1) head dipping, i.e., looking down from the edge of an open arm or the central platform; (2) rearing, i.e., standing up on the hind limbs with or without keeping the fore limbs on the wall and (3) grooming. Number of Closed arm entries and vertical rearing were measured as indication of exploratory activity of the rat. Videos were analyzed by observer blinded to the experiment.

### Novel Object Recognition (NOR) test

All the three groups were studied for recognition working memory. The rats were habituated to the empty box (100 × 50 × 50 cm) for 5 min over 2 days at the start of dark phase. The activities of the animals were recorded by a video camera. From the third day onward two objects having no smell but having different colors and shapes were placed in the boxes and rats were allowed to explore both the objects for 5 min daily up to 4 days. The objects were cleaned thoroughly with 5% ethanol to ensure the absence of olfactory cues. The exploration of object was defined by sniffing, licking, chewing by animals or by moving vibrissae while directing the nose towards the object within less than 1 cm distance. The number of episodes and time spent with each object was recorded and on the day of acquisition the least explored object was replaced with novel object. VSD and TSD rats were sleep deprived for 12 h and all the three groups animals were allowed to explore both the old and new objects for 5 min. The number of episodes and time spent in exploration of each object by each animal was recorded. Preference index for novel object (Time spent in exploration of novel object/Total time spent in exploration of both the objects) was calculated. Preference index greater than 0.5 was considered as higher preference for the novel object than the old one. Videos were analyzed by observer blinded to the experiment.

### Grooming behavior

We analyzed grooming behavior in all the three group which were kept to open field in novel object recognition to correlate the effect of stress and anxiety caused by SD on memory and cognition. The grooming behavior of rats was defined as licking and combing of various body parts including paw licking, snout/face grooming, head washing, body fur grooming with hind paws, leg and genital licking. Any grooming bout of duration more than 5 sec was considered as actual grooming bout. The number of grooming episodes and total time spent in grooming were recorded and averaged for each group.

### Rotarod Test

On the fifteenth day of TCE treatment, rotarod test was performed for all the three groups i.e., VUD, VSD and TSD. The apparatus was an automatic motor-driven treadmill (Rotamex-5; Columbus Instruments) consisting of a 7.0 × 9.5 cm spindle diameter with a fall height of 44.5 cm from the center. The rotarod test was performed on each animal for 5 min at a constant speed of 10 rpm. The number of falls and the time spent on the rotating rod were recorded for each trial and averaged for each group.

### Immunohistochemical analyses

Experimental animals (n = 3–4 each) were transcardially perfused with 4% paraformaldehyde (PFA) in 0.1M phosphate buffer saline (PBS). Brains were dissected out and incubated in the fixative (4% PFA) for overnight at 4 °C and subsequently cryopreserved in 20% and then 30% sucrose for 24 h each at 4 °C. Coronal sections (35 μm) were cut using cryostat microtome. Sections were incubated with 0.3% PBS-T (Triton-X 100) for 15 min, washed with 0.1% PBST thrice for 5 min each and incubated with 5% normal goat serum in PBS for 30 min, followed by 24 h incubation with respective antibodies (monoclonal mouse IgM PSA-NCAM (1:500) (Millipore, Bedford, MA, USA), mouse IgG GAP-43 (1:500) and rabbit IgG GFAP (1:500) (Sigma-Aldrich St. Louis, MO, USA)) at 4 °C. After incubation sections were washed with 0.1% PBST thrice for 5 min each and incubated with respective Alexaflour 488 labeled secondary antibodies diluted in 1XPBST for 2 h at RT. Tissue sections were washed thrice for 5 min each and then mounted on glass slides with Fluoromount (Sigma-Aldrich St. Louis, MO, USA). Images were captured by Nikon A1R Confocal Laser Microscope and analyzed using NIS elements AR analysis software version 4.11.00.

### Immunoblotting analyses

Animals (n = 3–4 each group) were sacrificed after anesthetizing by Thiopentone injection (1 unit per 10 g). Pyriform cortex (PC) and hippocampus regions were dissected out and lysed in lysing buffer (1X Tris Buffered Saline, DTT, Na_3_VO_4_, Protease inhibitor) using tissue homogenizer and centrifuged at 7000 rpm for 10 min at 4 °C. Supernatant was collected and protein concentration was estimated by Bradford method. 30–40 μg of protein from each sample was resolved in 7 and 10% SDS-PAGE followed by transfer onto a 0.45 μm pore size PVDF membrane using the semi-dry Novablot system (both from Amersham Biosciences UK Limited). After blocking with 5% skimmed milk in 1% TBS-Tween 20 for 2 h, membranes were probed with primary antibodies (mouse monoclonal anti-PSA-NCAM (IgM) (1:1000), mouse monoclonal anti-NCAM (1:2000), mouse monoclonal anti-GAP43 (1:2000), mouse monoclonal anti-CaN (1:2000), mouse monoclonal anti-mortalin (1:1000), mouse monoclonal anti-HSP-70 (1:2500), mouse monoclonal anti-bcl-xl (1:1000), mouse monoclonal anti-GFAP (1:2500), mouse monoclonal anti-NF-κB (1:2000), mouse monoclonal anti-TNF-α (1:1000), mouse monoclonal anti-IL-1β (1:1000), mouse monoclonal anti-IL-6 (1:1000), rabbit polyclonal anti-SNAP-25 (1:1000), rabbit polyclonal anti-pAkt-1^pser-473^ (1:2000), rabbit polyclonal anti-cyt. c (1:1000) (All from Sigma-Aldrich St. Louis, MO, USA), anti-NeuN (1:3000) (Chemicon International, Temecula, CA, USA), goat polyclonal anti-CamKII-α (1:2000), rabbit polyclonal anti-c-fos, rabbit polyclonal anti-c-jun (all from Santa Cruz Biotechnology, Inc.), rabbit anti-PARP antibody (from Invitrogen, Carlsbad, CA, USA), rat monoclonal anti-OX-42 (1:1000), rat monoclonal anti-OX-18 (1:1000) (both from BD Biosciences, San Jose, CA, USA) for overnight at 4 °C. Membranes were washed with 0.1% TBST and incubated with HRP-labeled respective secondary antibodies (from Merck Millipore USA) for 2 h at 25 °C. Membranes were washed thrice with 0.1%TBS-T and immunoreactive bands were detected by Amersham ECL Plus Western blot detection system (GE Healthcare Life Sciences UK Limted) using ImageQuant LAS 4000 (GE Healthcare Life Sciences UK Limited). Change in expression of protein of interest was the average of RDV values obtained from at least three independent experiments.

### Statistical analysis

Values were expressed as mean ± SEM of the values obtained from at least three independent experiments. The Sigma Stat for Windows (version 3.5) was used to analyze the results by Student’s *t*-test and one way ANOVA (Holm-Sidak post hoc method), in order to determine the significance of the mean values. Two-way ANOVA was performed on behavioral test results to determine the level of significance for the parameters in study, within the group and between the groups. Values with *P* value < 0.05 were considered as statistically significant.

## Additional Information

**How to cite this article**: Mishra, R. *et al*. *Tinospora cordifolia* ameliorates anxiety-like behavior and improves cognitive functions in acute sleep deprived rats. *Sci. Rep*. **6**, 25564; doi: 10.1038/srep25564 (2016).
